# Personalized Preoperative Prediction of the Length of Hospital Stay after TAVI Using a Dedicated Decision Tree Algorithm

**DOI:** 10.3390/jpm12030346

**Published:** 2022-02-24

**Authors:** Maria Zisiopoulou, Alexander Berkowitsch, Ralf Neuber, Haralampos Gouveris, Stephan Fichtlscherer, Thomas Walther, Mariuca Vasa-Nicotera, Philipp Seppelt

**Affiliations:** 1 Department of Cardiology, University Hospital Frankfurt, Goethe University Frankfurt am Main, 60590 Frankfurt am Main, Germany; alexander.berkowitsch@kgu.de (A.B.); ralf.neuber@kgu.de (R.N.); fichtlscherer@em.uni-frankfurt.de (S.F.); mariuca.vasa-nicotera@kgu.de (M.V.-N.); philipp.seppelt@kgu.de (P.S.); 2 Quality Management, Department of Otorhinolaryngology, University Medical Center Mainz, 55131 Mainz, Germany; haralampos.gouveris@unimedizin-mainz.de; 3 Department of Cardiothoracic Surgery, University Hospital Frankfurt, Goethe University Frankfurt am Main, 60590 Frankfurt am Main, Germany; thomas.walther@kgu.de

**Keywords:** prediction, patient-reported outcomes, aortic stenosis, TAVI, hospital length of stay, decision tree, algorithm

## Abstract

Background: The aim of this study was to identify pre-operative parameters able to predict length of stay (LoS) based on clinical data and patient-reported outcome measures (PROMs) from a scorecard database in patients with significant aortic stenosis who underwent TAVI (transfemoral aortic valve implantation). Methods: 302 participants (51.7% males, age range 78.2–84.2 years.) were prospectively recruited. After computing the median LoS value (=6 days, range = 5–8 days), we implemented a decision tree algorithm by setting dichotomized values at median LoS as the dependent variable and assessed baseline clinical variables and PROMs (Clinical Frailty Scale (CFS), EuroQol-5 Dimension-5 Levels (EQ-5D) and Kansas City Cardiomyopathy Questionnaire (KCCQ)) as potential predictors. Results: Among clinical parameters, only peripheral arterial disease (*p* = 0.029, HR = 1.826) and glomerular filtration rate (GFR, cut-off < 33 mL/min/1.73 m^2^, *p* = 0.003, HR = 2.252) were predictive of LoS. Additionally, two PROMs (CFS; cut-off = 3, *p* < 0.001, HR = 1.324 and KCCQ; cut-off = 30, *p* = 0.003, HR = 2.274) were strong predictors. Further, a risk score for LoS (RS_LoS) was calculated based on these predictors. Patients with RS_LoS = 0 had a median LoS of 5 days; patients RS_LoS ≥ 3 had a median LoS of 8 days. Conclusions: based on the pre-operative values of the above four predictors, a personalized prediction of LoS after TAVI can be achieved.

## 1. Introduction

Methods to help predict the post-interventional length of stay (LoS) may result in better and individualized decisions regarding the management of patients both during hospital stay as well as after hospital discharge. This predictive analytics methodology may have a significant impact on both patients’ and relatives’ expectations and informed consent and results in better time and resource (e.g., hospital beds) management. It may also improve communication among health professionals and patients or their relatives and therefore may enhance cooperation between patients/relatives and health care specialists.

Various classification algorithms, namely, Support Vector Machines (SVM), Artificial Neural Network (ANN), Naive Bayes, Linear Regression, Random Forest and Gradient Boosting have been already applied to predict length of hospital stay in various cardiac diseases or conditions. Decision Tree analyses have been applied previously to predict LoS in patients with coronary artery disease [1], after isolated coronary artery bypass graft surgery [2] or after acute type A aortic dissection surgery [3].

In addition, decision tree methods have also been used previously extensively in the prediction of non-cardiac conditions, such as pediatric and respiratory health conditions [4]. Using three different techniques to support the decision tree methodology, namely Bagging, Adaboost, and Random forest techniques Ma et al. (2020) [4] could very effectively predict LoS in pediatric and respiratory conditions.

In their comprehensive review of risk adjustment models of hospital LoS, Lu et al. (2015) [5] suggest that a promising approach to improve the performance of LoS predictive and risk adjustment models is to include more disease-specific variables, such as disease-specific or condition-specific measures, and functional measures. For such an approach, more comprehensive and standardized data are needed. Accordingly, in our present study we have concentrated on disease-specific, comprehensive and standardized data involving patients with severe aortic valve stenosis treated with TAVI (transfemoral aortic valve implantation).

Thus far, there have been no attempts to predict LoS in TAVI patients using classification algorithms. The aim of this prospective study was to find pre-operative parameters able to predict LoS based on clinical data and patient-reported outcome measures (PROMs) of a TAVI scorecard database.

## 2. Materials and Methods

Patients with severe aortic stenosis who were hospitalized and scheduled for a TAVI procedure and treated within 48 h after admission were included in the study. The study was designed as a prospective observational cohort study. The primary endpoint was post-procedural LoS. Patients who died in the acute post-TAVI period in hospital were excluded from analysis. Baseline anthropometric characteristics, medical conditions and comorbidities, biochemical parameters and PROMs were assessed on admission. All patients who underwent a transfemoral TAVI-procedure from January 2019 until April 2021 in the cardiology department of our tertiary university hospital were asked to participate in this prospective study.

It should be noted that, based on current reimbursement policies in our country, a LoS of less than 4 days after implantation would result in a reduction in reimbursement. Therefore, according to current practices, patients are discharged on the 4th postoperative day, unless complications occur.

### 2.1. Inclusion Criteria

Main inclusion criteria were a severe symptomatic degenerative aortic valve stenosis with an effective orifice area (EOA) < 1.0 cm^2^ or mean gradient > 40 mmHg and a NYHA (New York Heart Association) functional class equal or greater than II. The final decision to perform TAVI-implantation was made by the local Heart Team Board on an individual personalized basis.

### 2.2. Exclusion Criteria

Patients presenting with acute non-compensated cardiogenic shock or hemodynamic instability requiring inotropic support prior to TAVI, as well as patients with severe neurological disorders, dementia or inability to provide informed consent due to their mental condition were excluded. Furthermore, patients with an aortic prosthetic heart valve requiring a valve in valve procedure were not included.

All investigative procedures (i.e., PROMs, clinical scoring systems, and blood testing) were performed in accordance with the Declaration of Helsinki for studies in humans at admission to the hospital one day prior to intervention. Written informed consent was given by all participants. The study was approved by the Institutional Review Board (Ethics Committee of the Faculty of Medicine; Nr. 296/16).

### 2.3. Collected Data

#### 2.3.1. The TAVI Scorecard Database

In order to standardize TAVI-related data acquisition, we have conceived and implemented a TAVI scorecard. On this scorecard, we collected at baseline EuroSCORE II (ESII), comorbidities (see Table 1), blood hemoglobin, serum creatinine-, and N-terminal prohormone of brain natriuretic peptide (NTproBNP)-levels, estimated glomerular filtration rate (eGFR), as well as EuroQol-5 Dimension-5 Levels (EQ5D5L), Kansas City Cardiomyopathy Questionnaire (KCCQ) and clinical frailty scale (CFS).

The TAVI scorecard database has been implemented since 2019 in our tertiary cardiology department based on several features of a previous management system introduced by Kaplan and Norton [6]. The TAVI scorecard helps analyze the possible cause and effects of quantifiable, measurable values such as key performance indicators (KPIs) and targets for the clinical TAVI pathway. The TAVI scorecard measures KPIs from four perspectives, which include individual content and a high variation of targets. The four perspectives are the perspectives of the cardiologist in the sector of “Quality”, the perspective of the hospital staff in the sector “Process”, the perspective of the patients in “Outcome” and the perspective of the management in “Cost”. Quality improvement is a major topic of the TAVI scorecard, and one major goal in our University Hospital cardiology department is the reduction of the KPI LoS in TAVI patients.

#### 2.3.2. Anthropometric Data

Gender, BMI and age are presented on Table 1.

#### 2.3.3. Comorbidities

Clinically significant comorbidities are presented on Table 1.

#### 2.3.4. Laboratory Investigations/Biomarkers

Baseline eGFR calculated according to MDRD (Modification of Diet Renal Disease) formula blood hemoglobin, creatinine-, and NTproBNP-levels have been measured in samples obtained from the participants one day before the TAVI procedure.

#### 2.3.5. EuroQol-5 Dimension-5 Levels (EQ5D5L)

The international questionnaire EuroQol-5 Dimension-5 Levels (EQ5D5L) is a standardized measurement of the quality of life (QoL) of a patient [7]. It consists of questions that assess QoL on the current day. The first part involves five questions on mobility, self-care, usual activities, pain/discomfort, and anxiety/depression, defined in five dimensions (no/slight/moderate/severe/extreme problems) for any question. The second part is the EQ Visual Analog Scale, which is a self-rated health scale and ranges from 0 to 100, 100 means the best and 0 means the worst health you can imagine [8].

#### 2.3.6. Kansas City Cardiomyopathy Questionnaire (KCCQ)

The Kansas City Cardiomyopathy Questionnaire (KCCQ) is a sensitive, specific, and responsive health-related QoL measure for patients with heart disease [9,10]. The KCCQ is a 23-item, self-measurement instrument with four domains: 1. “Physical limitation” (PhyLi), 2. “Symptom frequency” (SymFre), 3. “Quality of life” (QoL) and 4. “Social limitation” (SoLi).

#### 2.3.7. Clinical Frailty Scale (CFS)

The CFS is a simple scale with a score ranging from 1 (very fit, performing sport activities on a regular basis) to 9 (terminally ill, with an estimated life expectancy of less than 6 months). The answers are based on descriptors and pictographs of activity and functional status. The patient is asked to tick in one of the nine boxes that describe their level of frailty in everyday domestic life [11,12].

#### 2.3.8. EuroSCORE II

EuroSCORE II is an established clinical scoring system to predict operative mortality from cardiac surgery. EuroSCORE II was determined prior to intervention for each patient with a web-based calculator (http://euroscore.org/calc.html). [13] However, EuroSCORE II does not include potentially relevant risk factors such as certain comorbidities (e.g., atrial fibrillation) or biomarkers such as blood levels of NTproBNP.

#### 2.3.9. Contrast Agent Volume

The volume of contrast agent used during the TAVI procedure in mL.

### 2.4. Intervention—TAVI Procedure

All participants were treated under conscious sedation. The TAVI procedure was performed as per standardized operating procedure protocols for balloon-expandable and self-expandable prostheses.

### 2.5. Statistical Analysis

The statistical analysis was performed as follows: first, we defined the median LoS value. Next, we performed a Decision Tree Algorithm (DTA) by setting dichotomized values at median LoS as the dependent variable and assessed baseline clinical variables and PROMs (Clinical Frailty Scale (CFS), EQ-5D and Kansas City Cardiomyopathy Questionnaire (KCCQ)) as potential predictors. The DTA allows identifying independent predictors and estimates their cut-off-values and their importance. The normalized importance of the independent predictors was determined by calculating the relative influence of each variable: whether that variable was selected to split on during the tree-building process, and how much the squared error (over all trees) improved (i.e., decreased) as a result. Whenever the model splits a node, based on a numeric or categorical feature, the feature’s attributed reduction in squared error is the difference in squared error between that node and its children nodes. The squared error for each individual node is the reduction in variance of the response value within that node [14].

A decision tree is a representation of a decision procedure for determining the class of a given instance. Each node of the tree specifies either a class name or a specific test that partitions the space of instances at the node according to the possible outcomes of the test. Each subset of the partition corresponds to a classification subproblem for that subspace of the instances, which is solved by a subtree. A decision tree can be seen as a divide-and-conquer strategy for object classification [15].

Further, predictors identified in the DTA were included in a multivariate binary regression model to estimate the hazard ratio (HR). Thereby, the continuous variables for which the DTA identified a respective cut-off value were further analyzed in a dichotomized mode. Given that many TAVI patients have various cardiac comorbidities, a possible impact of these comorbidities was analyzed separately as a merged variable (named “cardiac comorbidity”). This variable was equal to “1” if any cardiac comorbidity besides aortic stenosis was diagnosed on admission and equal to “0” in any other case. The data are reported as number (%) or median (IQR) where appropriate. The differences were considered significant by error probability *p* < 0.05.

## 3. Results

### 3.1. Baseline Data

A total of 302 patients who were discharged after TAVI were included; 19 of these patients (6.3%) died within 12 months after discharge. A number of anthropometric, managerial/procedural, clinical and biochemical data have been assessed (see Table 1). Median in-hospital post-TAVL LoS was 6 days and median ICU-LoS was 51 h. The baseline data are depicted on Table 1.

### 3.2. Analysis of LoS

The performed DTA (Table 2) identified eight variables as independent predictors of post-TAVI LoS. Four of the eight variables were also revealed in the binary regression. Interestingly, among diagnosed comorbidities, only PAD and renal dysfunction/renal failure were found to be predictive of LoS after TAVI. In addition, two PROMs (CFS and KCCQ) were also shown to be strong predictors.

**Figure 1:**
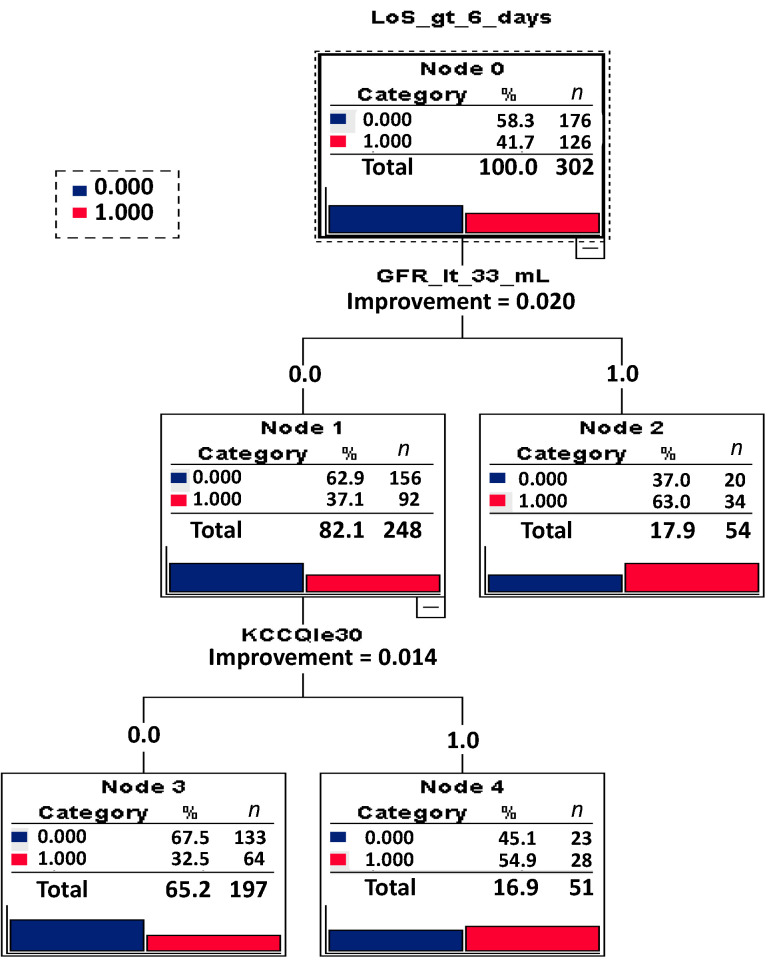
Decision Tree Algorithm for LoS > 6 days. Legend Figure 1. A decision tree is an algorithm that recursively divides the training data, based on certain splitting criteria, to predict a given target. This decision tree starts with all the participants in the root node (Node 0), then divides these participants into those with GFR less than 33 mL/min/1.73 m^2^, and those with GFR greater than or equal to 33 mL/min/1.73 m^2^; for all participants with GFR less than 33 mL/min/1.73 m^2^, an additional separation was made between participants with KCCQ-scores greater than 30 and participants with KCCQ-scores lower than or equal to 30. LoS_gt_6_days = LoS greater than 6 days; GFR_lt_33_mL = GFR lower than 33 mL; KCCQle30 = KCCQ score lower than or equal to 30. In each node, 0.0 (zero) means that the participants within this node do not have the respective feature (e.g., GFR_lt_33_mL) and 1.0 means that the participants do have this feature. Depicted with blue (or 0.000) are participants with LoS equal or lower than 6 days and depicted with red (or 1.000) are participants with LoS > 6 days.

The decision tree algorithm is depicted in Figure 1.

**Figure 2:**
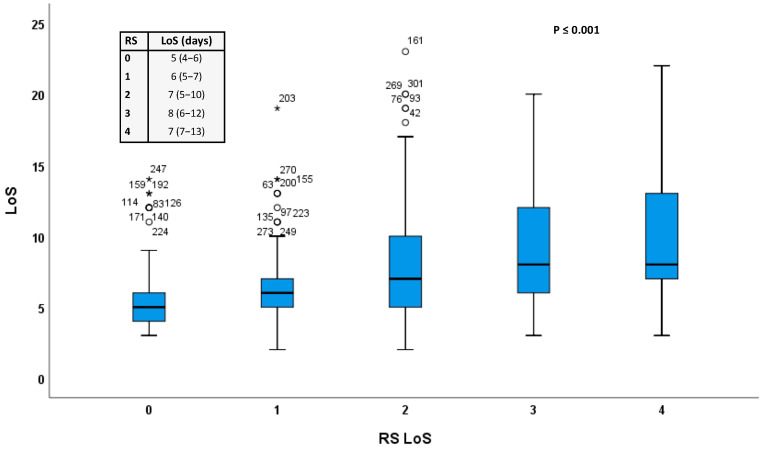
Distribution of LoS as a function of the calculated RS. Legend of Figure 2. Box plots depicting LoS (in days after TAVI), depending on the cumulative number of the aforementioned four predictors (RS_LoS range = 0−4). Within the box on the upper left corner of the figure the RS Score (0−4) is provided on the first column and the respective median numerical values, including the interquartile range within parentheses, are provided on the second column.

Further, based on the identified predictors of LoS> 6 days, a risk score (RS) for LoS (RS_LoS) was calculated by using a combination of the identified predictors. For example, a RS_ LoS = 2 was given to any patient who met two out of the four predictors that emerged after the binary regression analysis (i.e., GFR, KCCQ, CFS and PAD; see Table 2). Figure 2 demonstrates the difference in LoS resulting among increasing RS values. As it can be appreciated in Figure 2, patients with RS_LoS = 0 have a median LoS of 5 days, whereas patients RS_LoS ≥ 3 have a median LoS of 8 days.

## 4. Discussion

In the present study, we found that two clinical comorbidities, PAD and renal dysfunction, and two PROMs (CFS and KCCQ) were strong predictors of LoS after TAVI. These strong predictors were confirmed by means of both binary regression and DTA. Additionally, we provide the cut-off values of these predictors that can be used in a clinical setting to easily calculate the respective individual risk scores and hence estimate personalized LoS values for each TAVI patient preoperatively.

This method gives clinicians the opportunity to provide specific personalized information and consultation to TAVI candidates prior to the intervention. In this respect, the process of informed consent of the patients and their families/relatives is further individualized. Additional measures (e.g., post-TAVI rehabilitation) can better be prepared and planed according to the specific individual needs of each TAVI patient even before the TAVI procedure. Additionally, by filling in the questionnaires and analyzing their condition, the patients are involved in the whole process, which leads to a strengthening of patient participation and patient empowerment.

### 4.1. Strengths of the Study

This study has several strengths. It was a prospective study. Given that the study was conducted in a single medical center, there was no variability in the standard of provided care, an issue that could otherwise have confounded the results. The same group of clinicians/interventional cardiologists, with prior extensive experience, performed all transfemoral TAVI interventions. In addition, this is one of the few studies [16] with a prospective design to specifically assess the usefulness of preoperative PROMs in the calculation of LoS after TAVI.

Other authors have also previously stressed the relevance of frailty as a significant predictor of outcomes after TAVI. Albumin was found to be the most commonly used single-dimension frailty measure and the Fried or modified Fried phenotype were the most commonly used multidimensional frailty measures [17]. Frailty is being commonly defined by the presence of any three of the following five criteria: algorithm-defined grip strength, 15-foot walking tests, body mass index < 20 kg/m^2^, Katz activity of daily living ≤ 4/6, and serum albumin < 3.5 g/dl [11]. Some authors argue that frailty assessment should continue to be part of the preprocedural assessment to further improve patient outcomes after TAVI [18].

Different authors have been using other frailty indices according to Valve Academic Research Consortium-3 [19] recommendations (5-min walk test [5 MWT] and hand grip strength) as well as other available scales of frailty (Katz index, Elderly Mobility Scale [EMS], Canadian Study of Health and Aging [CSHA] scale, Identification of Seniors at Risk [ISAR] scale) at baseline as predictors of 12-month mortality [20].

Frailty was associated with worse outcomes following TAVI and incorporating frailty metrics significantly improved the predictive performance of existing clinical prediction models. Physician-estimated frailty measures could aid TAVI risk stratification, until more objective scales are routinely collected [21]. In another domain of cardiac interventions, namely percutaneous coronary intervention (PCI), simple assessment of frailty has been shown to help predict mortality and the length of hospital stay, and may therefore guide healthcare providers to plan PCI and appropriate resources for frail patients [22]. The impact of frailty on mortality, length of stay and rehospitalization in older hospitalized patients with atrial fibrillation has also been investigated [23]; frailty was associated with prolonged length of stay and increased mortality, but not re-admission during six months after discharge in atrial fibrillation patients [23].

In a recent scoping review of the Clinical Frailty Scale [18], mortality was the most common outcome examined with CFS being predictive 87% of the time. CFS was associated with comorbidity 73% of the time, complications 100%, length of stay 75%, falls 71%, cognition 94%, and function 91%. The CFS was associated with other frailty scores 94% of the time. In acute medical settings, the CFS helped identify patients that are more likely to have prolonged hospital stays. The CFS is an easy to use tool that can detect older adults at high risk of complicated course and longer stay. Objective early identification of seniors with frailty in acute care units can help to target interventions to prevent complications and to implement effective discharge planning in high risk older adults [24].

More specifically, regarding the specific CFS metric used in our present study, it has been shown that, in addition to reflecting the degree of frailty, the CFS is a useful marker for predicting late mortality in elderly transcatheter aortic valve replacement cohorts [11].

Nonetheless, frailty scores have been rarely used as predictors of LoS after TAVI. To the best of our knowledge, this is the first report using the CFS in a composite decision tree algorithm to predict LoS after TAVI. This fact also has implications, not only from a medical professionals’ and the patients’ perspective, but also from a managerial perspective;, e.g., within the framework of our TAVI-scorecard, the importance (weight) of the renal function—as depicted by GFR, CFS, KCCQ and peripheral arterial disease as key performance indicators is, respectively, increased. Accordingly, with such an evidence-based approach, all stakeholders can focus on these few key indicators, therefore minimizing the burden of meaningful data collection in everyday practice and using these key performance indicators to measure success of quality and safety improvement projects and protocols.

The importance of renal function as a major predictor of TAVI outcomes has also been documented in the relevant literature [25,26]. The strong relationship between significant aortic stenosis, TAVI-placement and renal function is further supported by the fact that over half of patients with compromised renal function who underwent TAVI showed an immediate improvement in kidney function post-TAVI [25]. Indeed, this effect appears to be TAVI-specific (e.g., when compared to percutaneous coronary intervention) and is possibly associated with favorable hemodynamic effects on renal perfusion after aortic valve replacement [27]. Severely impaired renal function (i.e., GFR < 30 mL/min) has showed a significant impact on 30-day (25%) and 12-month mortality (49%) [28].

Although even moderately reduced renal function at baseline (i.e., an estimated GFR ≤ 60 mL/min/1.73 m^2^) has been shown to be associated with a 14% rate of > 10% renal function decline [25], we show here that a much more significant functional decline (i.e., GFR < 33 mL/min/1.73 m^2^) actually has an impact on LoS.

A further argument in support of a strong association of TAVI-procedure and renal function is that acute kidney injury, defined as a reduction of >25% in estimated glomerular filtration rate (eGFR) within 48 h following the procedure or the need for hemodialysis during hospitalization, occurred in 11.7% of the patients following TAVI and was associated with a greater than four-fold increase in the risk of postoperative mortality [29].

The absence of use of contrast medium during TAVI placement, especially in patients with profoundly reduced GFR (i.e., <30 mL/min/1.73 m^2^) [30], may prove to be an essential factor influencing outcomes, including LoS.

As experience with the transfemoral TAVI placement accumulates in large-volume TAVI-centers, it will be interesting, as part of a future research agenda, to examine the influence of no-contrast procedures on LoS data. Moreover, the use of the aforementioned predictors in cohorts of low-risk aortic stenosis patients treated with TAVI [31] should be the focus of further studies. In addition, the need for standardization of frailty measurements in clinical everyday practice to promote reporting consistency has been already vividly highlighted with a meta-analysis [17].

Other authors found PAD in 31.3% of their TAVI patient cohort. TAVI-patients with PAD had higher incidence of major vascular complication (11.1% vs. 1.3%, *p* = 0.033) and 30-day mortality (13.9% vs. 1.3%, *p* < 0.001) [32]. In another study, TAVI patients with PAD, particularly those with critical limb ischemia (CLI), had a higher incidence of periprocedural stroke, bleeding and acute kidney injury (*p* < 0.001). The overall in-hospital mortality among TAVI without PAD, non-CLI PAD and CLI was 6.1%, 8.4% and 14.7%, respectively (*p* < 0.001). In a multivariate logistic regression analysis, CLI was an independent predictor of in-hospital mortality (*p* < 0.001) [33]. Although length of hospital stay was not a primary or secondary outcome in the aforementioned studies and, therefore, was not explicitly documented, it may be supposed that the above increased complication rates associated with PAD would have eventually lead to an increased in-hospital LoS.

We aimed from the beginning at creating an instrument which would be based on less technical data (e.g., LVEF, ECG) and more on easy-to-acquire patient-reported, historical (from the patients’ charts) and standardized clinical chemistry values. Of course, it makes sense to consider testing and integrating LVEF and pre-existing ECG abnormalities in such prognostic models in the future, since such features could play an important role in pathophysiological mechanisms, which could cause prolonged hospitalization in this patient group.

From a statistical methodological perspective the decision tree method has distinct advantages over other techniques. In a comparison of decision tree algorithms with linear and logistic regression, [34] suggested that unlike logistic and linear regression, decision tree does not develop a prediction equation. Instead, data are partitioned along the predictor axes into subsets with homogeneous values of the dependent variable—a process represented by a decision tree algorithm (DTA) that can be used to make predictions from new observations. We have used such a decision tree algorithm to identify powerful predictors. Additionally, we have defined adjusted hazard ratio in multivariate Cox regression. The parameters included in such a multivariate Cox regression should be selected either by significance in univariate regression or by selection in DTA. Using a DT algorithm results in a significantly reduced time burden.

### 4.2. Study Limitations

This was a pilot single-center study and should therefore be considered as hypothesis-generating. Accordingly, the present findings should be interpreted with caution and the respective DTA-algorithm should be further trained with more relevant TAVI patient data. Nonetheless, it should be noted that our post hoc power calculation resulted in a power of 83% for this patient sample size (302 patients). Furthermore, our study patients were implanted with TAVI devices from various providers/manufacturers (Abbott, Boston Scientific, Edwards, Medtronic) and hence, the specific implant type could have confounded the results. We did not analyze the impact of valve selection, and the rate of different valves on the rate of permanent pacemaker implantation (PPI) or other relevant complications that would probably prolong the hospital stay.

In this respect, it may also be useful in future studies to include pre-procedural electrocardiogram (ECG) findings suggestive of conduction disorders that may peri-operatively lead to PPI and hence prolong LoS.

Additionally, it should be noted that peri-procedural outcomes and events have not been included in our length of stay analysis. For instance, any issues concerning post-procedural conduction disturbances and the need of implanting a permanent pacemaker at the acute post-procedural setting (which, in a mixed setting of different types of valves like ours may be substantial) are not incorporated in our present model. If incorporated into such an analysis, these or similar factors may be significant cofounders, and therefore have a major impact on the results of the analysis.

## 5. Conclusions

This novel prospectively examined DTA method gives clinicians the opportunity to provide specific personalized information and consultation to TAVI candidates prior to the intervention. As a result, the process of informed consent of the patients and their families/relatives is further individualized.

## Figures and Tables

**Table 1:** Baseline characteristics of the study participants (number *n* = 302).

Baseline Characteristic	*n* Inter-Quantiles (Median) Range	% of Total Participants
Male sex, *n* of participants	156	51.66% of all participants
Age, years	81.27 (78.19−84.24)	-
ES II, score values	3.73 (2.18−6.40)	-
BMI, kg/m^2^	27.34 (25.37−29.58)	-
Post-TAVI LoS, days	6 (5−8)	-
ICU-LoS, hours	50.95 (46.82−74.67)	-
Mobilization time, hours	21.12 (9.67−26.17)	-
LVEF, %	50 (48−53)	-
CAD, *n* of participants	138	45.54% of all participants
CHF, *n* of participants	40	13.20% of all participants
Prior surgery, *n* of participants	28	9.24% of all participants
CS, *n* of participants	63	20.79% of all participants
PM/ICD implantation, *n*	34	11.22% of all participants
AF, *n* of participants	84	27.72% of all participants
Cardiac co-morbidity, *n*	185	61.06% of all participants
Aortic aneurysm, *n*	14	4.62% of all participants
PAD, *n* of participants	77	25.41% of all participants
DM, *n* of participants	65	21.45% of all participants
COPD, *n* of participants	46	15.18% of all participants
Neurological dysfunction, *n*	25	8.25% of all participants
Ranking scale score ≥ 2, score	5	1.65% of all participants
CFS, score	4 (3−6)	-
EQ-5D-5L, score	55 (38−77)	-
KCCQ, score	39 (30−48)	-
Hb, g/dL	12.60 (11.40−13.75)	-
GFR, mL/min/1.73 m^2^	48.70 (36.78−61.73)	-
NTproBNP, score	1569 (535.5−3623)	-
Contrast agent volume, mL	70 (50−90)	-

Baseline anthropometric, clinical, biochemical and patient-reported data of the study participants. Abbreviations: BMI: body mass index; LVEF: left ventricular ejection fraction; CAD: coronary artery disease; CHF: congestive heart failure; CS: history of fully compensated cardiogenic shock (>3 months before TAVI); PM/ICD: implanted pacemaker/cardioverter device; AF: atrial fibrillation; PAD: peripheral arterial disease, clinical significant PAD, namely ≥ 2 Fontaine stage and/or interventions on extremity arteries (pelvic, leg arteries) due to atherosclerosis have been performed or are planned; DM: diabetes mellitus; COPD: chronic obstructive pulmonary disease; Rankin scale score ≥ 2; CFS: clinical frailty scale; KCCQ: Kansas City Cardiomyopathy Questionnaire; EQ_5D: EuroQol; Hb: hemoglobin; GFR: glomerular filtration rate; ESII: Euroscore II.

**Table 2:** DTA and binary regression analysis for LoS.

Independent Variable	Importance	Normalized Importance	Cut-Off Values	*p*	HR	95% CI
**GFR**	**0.023**	**100.0%**	**<33 mL/min/1.73 m^2^**	**0.003**	**2.252**	**(1.316−3.852)**
NT pro BNP	0.016	69.9%		0.483	1.174	(0.750–1.840)
**CFS**	**0.015**	**64.4%**	**>3**	**0.000**	**1.324**	**(1.138−1.539)**
**KCCQ**	**0.013**	**55.6%**	**<30**	**0.003**	**2.274**	**(1.323−3.907)**
Hb	0.008	34.3%		0.808	1.008	(0.946−1.074)
Age	0.002	7.7%		0.385	0.987	(0.957−1.017)
BMI	0.001	5.0%		0.690	1.010	(0.961−1.062)
**PAD**	**0.012**	**60.6%**		**0.029**	**1.826**	**(1.062−3.140)**

HR was calculated for Log (NT pro BNP). HR for GFR, CFS and KCCQ were calculated for dichotomized modus. Independent variables that were statistically significant are printed in bold.

## Data Availability

Data supporting the reported results may be provided by the authors upon reasonable request.
